# Proceedings of the 9th Annual Dubai Medical Education Symposium 2015: Dubai, UAE. 30 May 2015

**DOI:** 10.1186/s12919-016-0002-4

**Published:** 2016-05-11

**Authors:** Fouzia Shersad, Hajer Sheikh, Munther I. Aldoori, Mohammed Galal El Din Ahmed, Joseph Michael Muscat-Baron, Dima Abdelmannan, Hossam Hamdy, Syed Tanzeem Haider Raza, Parveen J. Kumar, Fatheia Ali Bayoumy, Gloria Pelizzo, Mike Irani, Fouzia Shersad, Doaa Sultan, Sumaiah Ali Abdulwahab, Tahra AlMahmoud, M. Jawad Hashim, Margaret Ann Elzubeir, Frank Branicki, Bushra Parveen, R. Subha Parameshwaran, Shubhangi Pathak, Ghazala Khan, Sabeena Salam, Nabeerah Aftab, Tasneem Sandozi, Aksha Memon, Maira Khalid Mehmood, Nayyab Mohammed Tayyab Mustafa, Almas Parker, Kawther Hamdi, Sabeena Salam, Eman Abu-Gharbieh, Sabeena Salam, M. Jawad Hashim

**Affiliations:** Institutional Effectiveness, Dubai Medical College, Huddersfield, United Arab Emirates; Biochemistry Department, Dubai Medical College, Dubai, United Arab Emirates; Dubai Medical College, Dubai, United Arab Emirates; Calderdale & Huddersfield NHS Trust, Huddersfield, UK; Anatomy Department, Dubai Medical College, Dubai, United Arab Emirates; Dubai Medical College, Dubai, United Arab Emirates; Associate Clinical Dean, Dubai Medical College, Dubai, United Arab Emirates; Institute of Leadership in Higher Education, University of Sharjah, Sharjah, United Arab Emirates; Acute Medicine, Royal Bournemouth and Christchurch Hospitals, NHS Foundation Trust, Bournemouth, UK; Medicine and Education, Queen Mary University of London, London, UK; Pathology Department, Dubai Medical College, Dubai, United Arab Emirates; Department of the Mother and Child Health, Pediatric Surgery Unit, Fondazione IRCCS Policlinico San Matteo, University of Pavia, Pavia, Italy; Ashford & St Peter’s NHS Trust, Chertsey, UK; Institutional Effectiveness, Dubai Medical College, Dubai, United Arab Emirates; Department of Parasitology, Dubai Medical College, Dubai, United Arab Emirates; Clinical Skills & Simulation Center, King Abdulaziz University, Jeddah, Saudi Arabia; Department of Surgery, College of Medicine and Health Sciences, United Arab Emirates University, Al Ain, United Arab Emirates; Department of Family Medicine, College of Medicine and Health Sciences, United Arab Emirates University, Al Ain, United Arab Emirates; Department of Medical Education, College of Medicine and Health Sciences, United Arab Emirates University, Al Ain, United Arab Emirates; Library, Dubai Medical College, Dubai, United Arab Emirates; Department of Microbiology, K J Somaiya Medical College and Hospital, Mumbai, India; Dubai Pharmacy College, Dubai, United Arab Emirates; Dubai Medical College, Dubai, United Arab Emirates; Dubai Pharmacy College, Dubai, United Arab Emirates; Department of Pharmacology and Toxicology, Dubai Pharmacy College, Dubai, United Arab Emirates; Dubai Pharmacy College, Dubai, United Arab Emirates; Department of Family Medicine, UAE University, Al Ain, United Arab Emirates

## Abstract

I1 The 9^th^ Annual Dubai Medical Education Symposium 2015 addresses the recent strides in medical education and clinical practice

Fouzia Shersad, Hajer Sheikh

S1 Acute lower limb ischaemia — Snow White and the Seven "Ps"

Munther I. Aldoori

S2 Learning styles in medical education

Mohammed Galal El Din Ahmed

S3 Reflection and feedback in teaching in clinical settings

Joseph Michael Muscat-Baron

S4 Medical Education in the Millennial Era

Dima Abdelmannan

S5 Current trends in the assessment of clinical competency at the workplace

Hossam Hamdy

S6 Influence of culture in delivering education

Syed Tanzeem Haider Raza

S7 Medical education – its day-to-day value in caring for patient

Parveen J Kumar

S8 IgG-4 related diseases (IgG-4 RD): what the clinician needs to know

Fatheia Ali Bayoumy

S9 From fetus to infant: the pediatric surgical care come into its own

Gloria Pelizzo

S10 20 minutes to know rheumatic disease

Mike Irani

S11 Research trends at Dubai Medical College

Fouzia Shersad, Doaa Sultan

P1 The Clinical Skills & Simulation Center (CSSC): Facing challenges in implementing the standardized program during OSCE (Objective Structured Clinical Examination) for undergraduate medical students

Sumaiah Ali Abdulwahab

P2 Informed consent education in a multicultural medical environment: clinical clerks’ perspectives

Tahra AlMahmoud, M. Jawad Hashim, Margaret Ann Elzubeir, Frank Branicki

P3 Effectiveness of information literacy on the information retrieval activity of medical students

Bushra Parveen

P4 Opportunistic infections in AIDS

R. Subha Parameshwaran, Shubhangi Pathak

P5 From transactional to transformative learning: engaging early-stage students in community engagement activities

Ghazala Khan, Sabeena Salam, Nabeerah Aftab

P6 A study of drug utilization of over the counter (OTC) drugs in private pharmacies of a residential area of Dubai, UAE

Tasneem Sandozi, Aksha Memon, Maira Khalid Mehmood, Nayyab Mohammed Tayyab Mustafa, Almas Parker, Kawther Hamdi

P7 General education matters for envisioning holistic integrative thinking in healthcare professionals

Sabeena Salam

P8 Course report: multidimensional imaging and capturing the quality of offered courses

Eman Abu-Gharbieh, Sabeena Salam

P9 Recent advances in medical education

M. Jawad Hashim

## I1 The 9^th^ Annual Dubai Medical Education Symposium 2015 addresses the recent strides in medical education and clinical practice

### Fouzia Shersad^1^, Hajer Sheikh^2^

#### ^1^Institutional Effectiveness, Dubai Medical College, Dubai, UAE; ^2^Biochemistry Department, Dubai Medical College, Dubai, UAE

The 9th Annual Dubai Medical Education Symposium was a professional development opportunity for medical educators, held at the Mohammed Bin Rashid Academic Medical Center Auditorium at the Dubai Healthcare City under the patronage of Haj Saeed Ahmed Al Lootah. The ceremony was formally inaugurated by, Mr. Saleh Saeed Lootah, Vice Chairman, Executive Board, Dubai Medical College on May 30^th^, 2015.

This symposium has been hosted annually by Dubai Medical College (DMC) since 2005, as an official platform for disseminating knowledge on current medical education-related topics in the region. Being the first medical college in the country, the college has taken the lead to foster educators as its social accountability initiative through the Medical Research Fund.

The goals of the symposium are dissemination of best practices in medical education, professional development of doctors and healthcare professionals and fostering research and scholarship. Speakers from across the globe shared significant insights into the medical education. The abstracts of the speakers’ presentations and selected research papers have been compiled in this supplement.

The key highlight of this supplement is the original article by Prof. Munder El Doori portraying the novel concept of Acute Limb Ischemia in the background of “Snow White and the Seven P’s”.

Prof. Mohammed Galal explains the effects of learning styles on student learning, while Prof. Muscat Baron discusses the value of reflection in medical education. Prof. Parveen discusses the value of day to day life of a student in caring for patients and Dr. Tanzeem Raza explores the importance of culture in moulding their careers. Dr. Dima Abdel Mannan puts forward a whole new perspective on the divide between digital natives and digital immigrants in the millennial era while Prof. Hossam Hamdy focuses on workplace-based assessment.

Prof. Fatehia highlights what the clinicians need to know about the elusive IgG4 disease. Prof. Mike discusses rheumatic diseases and Dr. Gloria provides her take on pediatric surgery in Germany.

The theme for this year’s symposium included a research-based competition where prizes were distributed to the winners with exceptional research abstracts. All research papers were reviewed by reviewers for scientific soundness. Reviewers included experts from the fields of basic science, health science and medical education. Changes were made based on the reviewers’ recommendations. Out of 28 research papers received, 10 were selected to be included in this supplement, based on originality. The educational research papers touched upon curricular changes in medical and pharmaceutical education, roles of general education, information literacy and use of technology and standardized patients. In the field of health science, topics range from glomerular pathology and drug utilization to HIV.

We hope this supplement will provide an informative collection of speaker abstracts and research ideas which will trigger more researchers to explore them and widen the scope of research in medical education and medical sciences.

## S1 Acute lower limb ischaemia — Snow White and the Seven "Ps"

### Munther I. Aldoori^1,2^

#### ^1^Dubai Medical College, Dubai, UAE; ^2^Calderdale & Huddersfield NHS Trust, Huddersfield, UK

Acute limb ischaemia (ALI) is a common emergency that can lead to loss of life and limb if not treated swiftly. A vascular unit serving a community of half a million population can expect to treat approximately 75 such patients a year [1].

ALI is caused by thrombosis in 60 %, embolisation in 30 %, and in 10 % other causes, including; trauma, arterial dissection and popliteal artery entrapment in young and healthy muscular patients.

Thrombosis in the diseased segment of an artery is triggered by many factors, including rupture in an atheromatous plaque, hypovolaemia, hyperviscosity, thrombophilias, heart failure and occluded arterial by-pass graft.

More than 80 % of emboli originate from the left atrial appendages during atrial fibrillation; the others arise from variety of causes, including left ventricle in post-myocardial Infarction, heart valves in endocarditis (intravenous substance misusers), prosthetic metal valves with poor anticoagulation, synthetic bypass grafts, and paradoxical embolus, least common source is an atrial myxoma. Aneurysms can cause embolisation, but less frequently than one would expect, apart from popliteal aneurysms.

ALI is commonly mistaken for acute disc prolapse, Baker`s cyst, ruptured Achilles tendon and deep venous thrombosis. More serious is total failure to consider it particularly in young patients with no history of atrial fibrillation, smoking or other risk factors.

ALI can affect patients at any age [2] and should be considered in the differential diagnosis of all patients presenting with leg pain of sudden onset, irrespective of age and risk factors.

The current acronym “the six P’s”, (Pain, Pallor, Perishing cold, Pulselessness, Parasthesia, and Paralysis) summarizes the clinical presentation of ALI. However not all the six “Ps” need necessarily be present to establish the diagnosis. The mere presence of the first three P’s, sudden severe pain, pallor and coldness, should trigger alarm, as early diagnosis is paramount for better outcome.

The most urgent action is the involvement of the vascular surgeon and the administration of intravenous 5000 U of unfractionated heparin. This should be administered immediately, even if the patient is likely to be undergoing angiography or surgery. This is essential to prevent propagation of thrombosis to the small vessels which lead to high vascular bed resistance resulting in failure of subsequent interventions.

To emphasis the importance of administering heparin, I propose a new acronym which is easier to remember whilst also addressing the crucial issue of timely administration of heparin.

My acronym is Snow White and the Seven ("Ps"). Pain, Pallor, Perishing cold, Parasthesia, Paralysis and the new seventh (P) is the propagated thrombosis which should be prevented by early IV administration of heparin.

Vascular surgeon's decision is based on the stage they find the limb in.

If muscular power is present (patient can wiggle their toes) and sensation is intact, there is time to request arterial imaging. At this stage it is either managed by interventional radiology or surgery.

In an immediately threatened limb where muscular power is reduced but there is some sensation left, an emergency embolectomy is the answer.

Limb presenting with profound paralysis and anaesthesia on the dorsum of the foot and fixed mottled skin, is sadly unsalvageable. The option is to perform primary amputation to save patient's life. Remember, at the end of the day, life is more precious than a limb.

**References**

1. Callum K, Bradbury A. ABC of arterial and venous disease: Acute limb ischaemia. BMJ. 2000; 320(7237):764.

2. Brearley S. Acute leg Ischaemia. BMJ. 2013; 346: 2681

## S2 Learning styles in medical education

### Mohammed Galal El Din Ahmed

#### Anatomy Department, Dubai Medical College, Dubai, UAE

Information enters the brain through three main ways: sight, hearing and touch, the one used the most being called “Learning Style”.

There is a great degree of diversity among students in their preference for teaching styles. Visual Learners prefer to see information as pictures, while auditory learners prefer to hear information spoken. Tactile/Kinesthetic Learners prefer touch as their primary mode for taking in information. Though some learners have a preference for one of these learning modalities, 86.8 % of students have been reported to be multimodal [1].

Medical students in clinical and preclinical phases have shown to have a higher preference for kinesthetic perception compared to average students [2]. Preferences may shift if a student perceives this as necessary to master the learning objectives [3]. Dealing with patients may force the appearance of certain preferences in students.

No one learning style is better than the other. Knowing about each learning style will help teachers to better understand how students learn and how to differentiate instructions. Therefore, medical education has shifted from didactic teaching to the use of interactive, problem-based, student-centered learning.

There is no evidence that instructors succeed in improving learning by adjusting instruction to the individual student’s learning style. Intrinsic motivation and prior knowledge are the only items proven by research to have any predictability of student success [4].

Many educational/cognitive psychologists believe learning styles are a myth, and that while individual differences in learning exists due to acquired/innate preferences they do not affect learning any more than the truck delivering groceries to your local store affects your dietary habits. We should consider that some ways of teaching are better suited to some subjects despite the learning styles of individuals.

**References**

1. Prithishkumar IJ, Michael SA, et al. Understanding your student: Using the VARK model. J Postgrad Med. 2014;60(2):183.

2. Ahmed MG, Gawish SM, Khalil MM. Toward better understanding of Medical students: Learning Styles of preclinical and clinical Medical Students. International Conference in Medical Education 2010. Abu Dhabi, UAE.

3. Pinto JK, Geiger MA, Boyle EJ. A three-year longitudinal study of changes in student learning styles. J Coll Stud Dev [Internet]. 1994 [cited 2015 Nov 15]; Available from: http://psycnet.apa.org/psycinfo/1994-47046-001

4. Holden JT, Westfall P. Learning Styles: Implications for Instructional Design. International journal of instructional technology and distance learning. 2011; 8(6).

## S3 Reflection and feedback in teaching in clinical settings

### Joseph Michael Muscat-Baron

#### Dubai Medical College, Dubai, UAE

**Introduction**

Feedback is the cornerstone of effective clinical teaching. Learners need feedback because it stimulates them to improve and provides information to learners with the intention of narrowing the gap between actual and desired performance. It also encourages learners to think about their performance and how they might improve it.

This talk will describe the principles of feedback, barriers to giving feedback and the negative effects of not giving feedback.

**Review of literature**

The concept of feedback is strongly theory based and it is shown to strongly reinforce knowledge and modify behavior. Cognitive theory shows that feedback helps learners to reconstruct their knowledge or thus modify their behavior. Feedback, if not carefully managed can results in demotivation and deterioration of performance. Feedback and assessment are closely related educational activities.

**Relationship between feedback and assessment**

Assessment is often described as a continuum between “formative” and “summative” assessment.

- “Formative” assessment essentially provides feedback in order to support and enhance learning and thus alter behavior. Intent is to share information about actual performance.
- “Summative” assessment is about measuring learners’ achievements with the purpose of grading or informing decisions about progression. Intent is more about conferring judgment rather than affecting the required change in behavior.

**The Techniques of providing feedback**

- 1 - The feedback - sandwich.
- 2 - The Pendleton model
- 3 - The reflective - feedback – conversation

**Conclusions**

- Feedback is fundamental to effective teaching and supervision
- Feedback is often absent or inadequate.
- Without feedback, good performance is not reinforced and poor performance can be repeated.
- Properly handled, feedback enhances teacher-learner relationship and leads to changes in learner’s behavior.
- Giving feedback can be learnt by repeated practice and by reflection on one’s performance.

## S4 Medical Education in the Millennial Era

### Dima Abdelmannan

#### Associate Clinical Dean, Dubai Medical College, Dubai, UAE

In 2001 Marc Prensky developed new vocabulary to characterize learners according to the influence of digital technology on the person’s behavior. Those born after 1980 (or thereabouts) were termed digital natives (also known as millennials or Generation Y), whereas those born before 1980 were termed digital immigrants. Some of the characteristic features of digital natives are: They read in an "F" pattern rather than in a "Z" pattern of digital immigrants. Moreover, digital natives tend to “dive in” only if the topic they were reading is interesting to them instead of reading the whole material. Digital natives use social media abundantly. They prefer to receive information quickly, to reply to that information instantly, and to receive information from multiple sources. In order to be able to receive information quickly and from multiple sources, the human brain compensates by decreasing the extent of information processing. Therefore, digital natives process information sub optimally. These traits are in sharp contrast to those of digital immigrants. Digital natives were brought up surrounded by digital media; as such their brains and thought processes are wired in a way different from the brains of digital immigrants who were brought up before the advent of technology.

Such a contrast of traits between digital natives and immigrants, raises the debate on overhauling education system in order to teach digital natives. However, radical changes in any field are not a solution to any problem. Purposeful teaching is a characteristic trait of humans which has not been seen in any other species in animal kingdom. Definitely, the field of teaching and learning has certain enduring qualities that have little or nothing to do with technology. As such, educators need to rethink on these enduring qualities in order to promote teaching and learning for digital natives, rather than focusing on any radical change in education system.

**References**

Jones Q, Ravid G, Rafaeli S. Information Overload and the Message Dynamics of Online Interaction Spaces: A Theoretical Model and Empirical Exploration. *Information System Research.* 2004; 15(2): 194–210.

Marc Prensky. Digital natives, digital immigrants. *On the Horizon.* 2001; 9(5).

Marc Prensky. Do they really *think* differently? *On the Horizon*. 2001; 9(5).

## S5 Current trends in the assessment of clinical competency at the workplace

### Hossam Hamdy

#### Institute of Leadership in Higher Education, University of Sharjah, Sharjah, UAE

The presentation highlights the current trends in assessment of clinical competencies particularly at the workplace. The training of health professionals has changed from unstructured, gaining experience by “osmosis” and difficult to assess, to structured, gaining experience by coaching and measuring achievements in real life practice environment. To assess competency, it is important to understand what clinical competency as a concept is, how it is acquired and how best it could be assessed.

The presentation will pull on lessons learnt from experiences and research on assessment of professional competency and problems. It will challenge some common practices, particularly deconstructing competence and measure its components e.g. OSCE.

The presentation supports the importance of global assessment, close observation of the trainee, the importance of feedback and how to combine formative and summative assessment “a fusional model”, and support the importance of the holistic judgment and qualitative value in assessment of clinical competency at the workplace.

## S6 Influence of culture in delivering education

### Syed Tanzeem Haider Raza

#### Acute Medicine, Royal Bournemouth and Christchurch Hospitals, NHS Foundation Trust, Bournemouth, UK

Culture plays a major role in an individual person’s behavior in any situation. It also has a major impact in how one may learn or respond to teaching. Although we all talk about ‘culture’ it is important to understand what it means in every day conversation. In my presentation, I aim to define culture in the broadest sense as “a way of life of a group of people – their behaviors, beliefs, values and symbols that they accept and are passed along by communication and imitation from one generation to the next”.

Culture can be explained as the ‘collective programming’ of brain in a given society. It is important to understand that usually there are several layers of culture even within a given society or a group of people.

My presentation explores various measures that can be used to identify specific characteristics within a society as determined by culture. These measures include concepts like social ‘power’ distance; individualism vs. collectivism; ability to deal with uncertainty and long-term vs. short term orientation towards life in different cultures.

Once we understand these fundamental concepts about culture, we can unpick how these habits and values can affect an individual’s learning and behavior in the class room. For example in some cultures all education has to be ‘teacher centred’ and effective teaching would depend entirely on how ‘good’ an individual teacher might be. These cultural backgrounds would flourish better if the students are given a strict timetable or a structure for their activities. Contrasting that with more ‘Westernised’ culture would suggest that the students might prefer more freedom in their learning; they might become more self-directed and might respond better to problem based learning with their own initiative rather than following a very rigid schedule or a timetable.

Some examples from personal experience are used to show how their cultural background had impacted on the progress of trainees who came to UK for their medical training.

Teachers in a multi-ethnic society need to be aware of the different dimensions and layers of culture and to appreciate how deeply these cultural values might be embedded in any individual. It is important to be conscious of these issues while helping trainees/students achieve their full potential.

## S7 Medical education – its day-to-day value in caring for patient

### Parveen J Kumar

#### Medicine and Education, Queen Mary University of London, London, UK

Medical education has a gone a long way from the time that Flexner introduced far-reaching changes to the American and Canadian system. Over the years, with changing curricula, medical education has become much more focused on the patient with emphasis on the clinical and communication skills for a good doctor-patient relationship. However, this is not a substitute for a good sound knowledge base which sometimes seems to be side-lined in the training to be a ‘nice’ doctor!

The need for early introduction of clinical exposure has been stated to be the single most important factor to achieving success in the current education system. Priority has therefore been given to non-medical subjects like professionalism and ethical behaviors, over the last decade.

The current focus is on the creation of holistic personal traits to produce a well-rounded individual. Inclusion of Arts, humanities and even business skills in medical education should be viewed seriously. The lack of critical thinking skills among the new generation has been countered by a variety of pedagogical innovations. Assessment system has been moving from subjective methods to standardized, feasible and practical methods. As we move further into the world of simulation in learning and assessment we need to sit back and reconsider the core objectives of the medical education curriculum.

Globalization has highlighted the need for international cooperation to help in the transmigration of medical professionals from one continent to another. Diseases will spread with the relative ease of travel across the world. It is therefore imperative that we train doctors for the ‘world’ and not just for a particular country’s medical problems. However, each country has its own credentialing system which doctors need to pass to practice.

With modern rapidly increasing innovations in technology, there is a danger that the focus will shift from the patient to a more utilitarian approach of the greatest good for the greatest number. However commendable utilitarianism might be, I hope we have enough sense for this not to happen!

## S8 IgG-4 related diseases (IgG-4 RD): what the clinician needs to know

### Fatheia Ali Bayoumy

#### Pathology Department, Dubai Medical College, Dubai, UAE

Since Immunoglobulin G4-related disease (IgG4-RD) has a wide variety of clinical presentations, this tends to pose difficulty to the clinician in his day-to day practice. It can be pathologically described as a systemic fibro-inflammatory disorder. Though this condition was known to exist in the last century, it was clinically and histopathologically characterized as a systemic illness only in the recent past.

One of the common examples of IgG4 related Disease is Type 1 autoimmune pancreatitis though other sites such as the orbit, retroperitoneum and salivary glands can also be involved (Pusztaszeri et al., 2012) (Ishida Fushiki and Watanabe, 2011).

The classical histopathologic appearance is that of excessive proliferation of lymphocytes and IgG4-secreting plasma cells in the background of storiform fibrosis and thrombophlebitis. (Deshpande, 2012) Since Serum levels of IgG4 may not be raised in some patients with classical IgG4 disease, diagnosis is based on retrospective analysis of clinical presentation. Therefore, several diagnostic criteria have been proposed. (Markus et al., 2012) Treatment is with glucocorticoids and adjunctive immunosuppression with drugs like Rituximab in refractory cases.

This presentation aims at providing clinical scenarios drawn from cases which can be easily missed. This is of paramount importance as it is a treatable condition which can be diagnosed by histopathology and therefore, warrants a high index of suspicion from the clinicians.

**Keywords***IgG4-RD, IgG4/IgG ratio, Storiform fibrosis, Lymphoplasmacytic inflammation, obliterative phlebitis*

**References**

Deshpande V. The pathology of IgG-4 related disease: critical issues and challenges. *Semin Diagn Pathol.* 2012; 29(4): 191–196

Ishida M, Fushiki H, Watanabe Y. An IgG4-Related Salivary Gland Disorder: A Case Series Presenting with a Different Clinical Setting. *Case Reports in Immunology.* 2011; 2011

Pusztaszeri M, Triponez F, Pache JC, Bongiovanni M. Riedel's thyroiditis with increased IgG4 plasma cells: evidence for an underlying IgG4-related sclerosing disease? *Thyroid*. 2012; 22(9): 964–968

Stone JH, Zen Y, Deshpande V. IgG4-Related Disease. *N Engl J Med*. 2012; 366: 539–551

Yaqiong L, Zhou G, Ozaki T, Nishihara E, Matsuzuka F, Bai Y, et al. Distinct histopathological features of Hashimoto’s thyroiditis with respect to IgG4-related disease. *Modern Pathology*. 2012; 25: 1086–1097

## S9 From fetus to infant: the pediatric surgical care come into its own

### Gloria Pelizzo

#### Department of the Mother and Child Health, Pediatric Surgery Unit, Fondazione IRCCS Policlinico San Matteo, University of Pavia, Pavia, Italy

Pediatric surgeons specialize in the surgical care of children and understanding their special needs. By training, they are oriented toward working with children and various specialists who are also oriented towards the next century. Pediatric surgeons are able to save whole lifetimes, and have the opportunity to follow their patients through a productive young life into adulthood.

Although relevant diagnostic and therapeutic tools have been progressively improving in the last decades, contributing to a new understanding of the natural history and physiologic outcomes of the congenital malformations, new frontiers in pediatric surgery (miniinvasive techniques, cell therapy, tissue engineering) are required in order to modify and ameliorate the treatment of congenital and acquired pathologies.

Quality of care in pediatric surgery could benefit from the following steps

- Planning of the pediatric surgery basic care and more demanding surgical skilling (pediatric robotic surgery from neonatal period to adolescents) under a teaching perspective of learning.
- Continuing education program for a well-trained team-work.
- Obtaining a best knowledge of the natural history of congenital malformation as the first step to develop new fields in pediatric research
- Defining a pediatric surgeon's road to research independence as the innovative vision in pediatric fields. Planning pediatric research program is needed in prenatal, neonatal and adolescent pathologies to obtain a best pediatric surgical care for a better life expectancy of the affected children; management of pediatric chronic diseases which seems to show a rising rate and children’s health and environment evaluation; development of new horizon of pediatric tissue engineering (stem cells); development of dedicated devices for innovative and invasive less techniques

The Multidisciplinary approach in Pediatric Surgery could be considered as a model of treatment which improve the quality of care. Infrastructure is also associated with better health outcomes, lower costs, and a more equitable health care system.

Although all the changes in our pediatric health care system, one thing remains constant: the needs of a sick child continuing relationship with relatives. Doctor has to act as an advocate to help him.

## S10 20 minutes to know rheumatic disease

### Mike Irani

#### Ashford & St Peter’s NHS Trust, Surrey, UK

**Introduction**

Unfortunately, despite the prevalence of rheumatic diseases, the subject is poorly taught as the subject matter tends to be under the remit of orthopaedic specialists and now musculoskeletal doctors. Also, the advance of immunology techniques and therapies has divided rheumatology into mechanical and auto-immune disciplines.

It is hoped that by simplifying, without losing accuracy, that the subject matter and diagnosis can be simplified according to the basic principles of history, examination and investigation.

**The essence**

Adhering to presentation of a patient, as is well recognised, 90 % of a diagnosis is made by the history. The questions to be asked are:

- The essential symptoms
- Date of onset
- Speed of onset
- Response to therapies prior to presentation

Following that, the cardinal features of inflammation, of swelling, redness, warmth, pain and loss of function should be ascertained and particular notice taken of duration of early morning stiffness or post-inactivity stiffness.

The past medical history and familial conditions which would in part a hereditary effect must be ascertained. Social habits of smoking and work, domicile and travel are vital.

Clinical examination should involve not only a directed inspection of the affected sites but also a general medical examination.

Investigations would revolve around those effects that are most likely to occur or to be found in the presence of an inflammatory condition.

**Conclusion**

It is hoped that by having a simplified taxonomy of disease presentation, signs on clinical inspection and confirmation by laboratory and imaging techniques would either confirm or at least point towards putative diagnosis.

I hope by simplifying the mystique of rheumatology will be less of a conundrum for non-specialists.

## S11 Research trends at Dubai Medical College

### Fouzia Shersad^1^, Doaa Sultan^2^

#### ^1^Institutional Effectiveness, Dubai Medical College, Dubai, UAE; ^2^Department of Parasitology, Dubai Medical College, Dubai, UAE

From early 1900s we have seen the emphasis of research move from basic research to applied research. With the emergence of the ‘Research and Development’ concept, the interest shifted to demonstrating the tangible public benefit in order to justify the billions of dollars spent on these activities. It is widely recognized that globally, the thrust for research follows the principles of demand forces and supply forces. The industry’s thrust should be balanced by the real needs of the society.

Scholarship in medical education is considered to be for the purpose of improving education as proposed by Ernest Boyer in 1990. The purpose of educational research is either to justify, clarify or describe as shown by description, justification and clarification studies. Research being the core purpose of an academician, a mandate on performing research could be inhibitive rather than encouraging. It is desirable to make it a journey of learning, answering questions and contribution to scientific knowledge. Therefore, medical education research should focus more on clarification (how and why) studies.

At DMC, research funding introduced professional development and encouragement of research. Funding was provided by internal; sources such as the medical research fund Medical Research Fund (MRF), which was set up by the founders to promote research in the whole Gulf region. It was seen that a total fund of 30,000 AED has been allotted. 8 DMC faculty members have utilised this fund and a total of 11 research proposals have been approved. 9 external researchers were also included in the program. Trends from 2003 showed a steady increase with a peak in 2010 and 2015. A shift towards full paper presentation was also observed over the years.

In conclusion, we see that educators should strike a balance between the demand and supply forces as the core purpose of research is to serve the community.

**References**

Albert M, Hodges B, Regehr G. Research in Medical Education: Balancing Service and Science*. *Advances in Health Sciences Education*. 2007; 12(1): 103–115.

Boyer EL. Scholarship Reconsidered: Priorities of the Professoriate. Princeton, NJ: Carnegie Foundation for the Advancement of Teaching, 1990.

## P1 The Clinical Skills & Simulation Center (CSSC): Facing challenges in implementing the standardized program during OSCE (Objective Structured Clinical Examination) for undergraduate medical students

### Sumaiah Ali Abdulwahab

#### Clinical Skills & Simulation Center, King Abdulaziz University, Jeddah, Saudi Arabia

**Introduction**

To ensure a high-quality undergraduate program, The Faculty of Medicine in 2011, had implemented a well-structured standardized program after intensive faculty training through which faculty obtained fellowship from University of Illinois in Chicago (UIC).

**Aim**

The aim of this study is to explore if the implementation of standardized patient (SP) program during the summative assessment (OSCE) had improved student outcomes and to find out the limitations of SP program.

**Methods**

A Survey consisting of eight questions were distributed to 125 medical students in 4th and 5th year. Most of the questionnaires were about SPs performance. These SPs were students who had been screened, recruited and hired from other colleges on campus. They were given onsite training on the day of the exam, to avoid disclosure of exam questions.

**Results**

58 % to 68 % of medical students (MS) felt that the appearances of SPs did not fit portrayed cases, which were mostly aged over 40, and so cases lacked reliability and validity. 53 % to 63 % of MS stated SPs were supportive and cooperative, however, 50 % to 60 % declared that SPs were not well trained. MS requested for trained SPs during their clinical teaching sessions and formative assessments. 63 % to 73 % of MS stated that having their OSCE at CSSC with Audio/Visual technology had controlled the flow and had organized the OSCE.

**Conclusion**

The SP program is an important tool to practice, in safe environment, non-technical skills such as patient interviewing, communication skills, breaking bad news and physical examinations. For the success of SP program, all portrayed cases must be validated by the curriculum committee and should be implemented in Formative OSCE for all undergraduates to improve the outcomes.

**References**

1. Boulet JR, De Champlain AF, McKinley DW. Setting defensible performance standards on OSCE and Standardized Patient examinations. *Medical Teacher.* 2003; 25(3): 245–249.

2. Carline JD, Paauw DS, Thiede KW, Ramsey PG. Factors affecting the reliability of rating of students' clinical skills in a medicine clerkship. *J Gen Int Med.* 1992: 7(5): 506–510.

3. Tekian A. & Yuskowsky R. (2009). Oral Examination (chapter11) in Assessment Methods in Health Professions Education by Downing S.M & Yuskowsty R. (Edu) New York and London: Roulodge.

## P2 Informed consent education in a multicultural medical environment: clinical clerks’ perspectives

### Tahra AlMahmoud^1^, M. Jawad Hashim^2^, Margaret Ann Elzubeir^3^, Frank Branicki^1^

#### ^1^Department of Surgery, College of Medicine and Health Sciences, United Arab Emirates University, Al Ain, UAE; ^2^Department of Family Medicine, College of Medicine and Health Sciences, United Arab Emirates University, Al Ain, UAE; ^3^Department of Medical Education, College of Medicine and Health Sciences, United Arab Emirates University, Al Ain, UAE

##### **Correspondence:** Tahra AlMahmoud - Department of Surgery, College of Medicine and Health Sciences, United Arab Emirates University, Al Ain, UAE

**Purpose**

Ethics training and professionalism theme exposure varies across curricula. The authors were interested in exploring medical students’ perceived need for educational training about informed consent and other ethical issues in a developing cosmopolitan country with unique cultures.

**Methods**

An anonymous paper survey, adapted from a published study, was distributed to 128 final year clinical clerks. The survey items addressed the level of educational attention needed for specified ethics topics compared to the amount currently provided.

**Results**

84 % completed the survey. Students indicated need for more attention to all topics related to informed consent (mean = 7.07 ± 1.24, on a scale of 1 to 9). Most additional attention was requested for topics discussing risks, benefits and alternatives to the recommended treatment with patients (7.34 ± 1.44), conducting assessments of decision- making capacity (7.29 ± 1.67), obtaining informed consent or refusal from surrogate decision-makers (7.08 ± 1.57), conducting assessments of decision-making capacity (7.29 ± 1.67), and obtaining informed consent from patients who are capable of making decisions (7.00 ± 1.69). The cohort expressed need for care of vulnerable patients’ education (7.24 ± 1.19) with maximum score for care of abused children. Women perceived greater curricular needs for many items of ethics education than did male respondents (p > 0.05). There were significant differences between students who scored high or low on the item being treated in an ethical professional manner and endorsement of educational needs for care of adolescents (mean 6.65 ± 1.72 versus 7.26 ± 1.35 respectively, p = 0.048).

**Conclusion**

There was high perception of need for more academic attention to all topics pertaining to ethical issues surrounding informed consent and educational care of vulnerable patients.

## P3 Effectiveness of information literacy on the information retrieval activity of medical students

### Bushra Parveen (bushra@dmcg.edu)

#### Library, Dubai Medical College, UAE

What started as a Library Orientation grew as a Bibliographic Instruction and finally became Information Literacy, which is the ability to identify what information is needed, understand how the information is organized, identify the best sources of information for a given need, locate the sources, evaluate the sources critically, and share the information.

Information Literacy is highly significant in today’s scenario as the mission of higher education institutions is the development of lifelong learners now, who continue to learn beyond their formal education. If the students specially the medical ones who will be the healthcare providers in their near future are able to reason and think critically and learn how to learn, they will be able to continue to grow intellectually throughout their careers and contribute to the society as informed citizens.

So to know the effectiveness of ‘Information Literacy Sessions’ a survey using descriptive research method has been done on the medical Students of ‘University of Sharjah’, UAE, where both ‘General’ and ‘Assignment based’ sessions are going on. The survey was done through the questionnaire given to the medical students visiting ‘Medical Library’ of ‘University of Sharjah’. The survey study will definitely clear the dilemma of implementation of these sessions in any medical institution.

During the interpretation of the data it was found that 72.5 % of the participating students always attend the ‘Information Literacy Sessions’ going on during their course time to time. 100 % of the students who attend the sessions feel that they get appropriate information about the library resource during their session. 89.65 % of the students understand most of the part of the session. 100 % students admitted that they keep in mind the instruction they get during their session while searching information in the library and the instruction really helps in the process to get accurate information. 89.65 % students said that the ‘Assignment based Information Literacy Session’ makes their assignment more effective and 79.31 % of students feel that they will be lost without attending these sessions.

So the data interpretation of the survey is clearly showing the importance of ‘Information Literacy Sessions’ in medicine during the course and also clears the dilemma of implementation of the sessions in the curriculum.

## P4 Opportunistic infections in AIDS

### R. Subha Parameshwaran, Shubhangi Pathak

#### Department of Microbiology, K J Somaiya Medical College and Hospital, Mumbai, India

**Introduction**

Opportunistic infections are common manifestations in AIDS. The type of infection depends on the immune status of the patient. The lowering of immunological status leads to decrease of helper cells. The common infection varies in different countries. Knowledge of common infections in AIDS helps in early diagnosis and treatment. Hence the prevalence of opportunistic infection was studied, in seropositive patients.

**Material and methods**

150 seropositive patients of Human immunodeficiency virus, suspected with infections were screened. The sputum, stool, respiratory fluid, cerebrospinal fluid and urine of patients were collected. Microscopy, culture and sensitivity were performed on all the samples, the isolates were identified by bio typing and serotyping. The tests were performed as per standard protocol and the results were analysed.

**Results**

87 (58 %) out of 150 samples were positive. 30 (34.4 %) samples were positive for mycobacterium, 25 (28.7 %) were positive for Candida, 11(12.6 %) samples showed Cryptococcus, 8 (9 %) for pneumocystitis jiroveci, 8 (9 %) for cryptosporidium, and 5 (5.7 %) for Isospora.

**Conclusion**

Mycobacterium and candidiasis were found to be the most common infections. Early diagnosis helps the clinician to initiate therapy. It can reduce the morbidity and increase life expectancy of the patient.

## P5 From transactional to transformative learning: engaging early-stage students in community engagement activities

### Ghazala Khan, Sabeena Salam, Nabeerah Aftab

#### Dubai Pharmacy College, Dubai, UAE

##### **Correspondence:** Ghazala Khan - Dubai Pharmacy College, Dubai, UAE

Societal needs and personal development of learners can be addressed through utilization of suitable community engagement activities in educational environments. At present, predominantly in Higher Education, transformative learning through community engagement is much in news, as part of the solution to many issues presently facing the community. At large, the learning transaction involves five kinds of “conversations” between teacher and learner: telling, showing, asking, responding, and giving feedback. Participation in community engagement activities encourages transformative learning that is stated as “the process of using a prior interpretation to construe a new or revised interpretation of the meaning of one’s experience in order to guide future action” (Mezirow, 1991). This poster describes a case study of initiatives taken by the community engagement unit with responsibility for introducing structured community engagement activities embedded within the academic calendar of the institution. A range of community engagement activities blended with social, educational and environmental domains as reusable patterns with wider applicability is deployed. Results indicated that degree of participation in such group work activities is directly related to a noteworthy positive transformative behavioral change among the students leading to a number of developmental outcomes in the following areas:

1. Personal Outcomes: Greater sense of personal efficacy, personal identity, and moral development and interpersonal development, particularly the ability to work well with others, and build leadership and communication skills.
2. Social Outcomes: Reduced stereotypes and greater inter-cultural understanding, improved social responsibility and citizenship skills, greater involvement in community service as lifelong learners.
3. Career Development: Improved academic learning, leadership skills, and personal efficacy which will lead to greater opportunity.
4. Relationship with the Institution: stronger relationships with faculty and greater satisfaction with college.

**Keywords:** community engagement, transactional, transformative learning

## P6 A study of drug utilization of over the counter (OTC) drugs in private pharmacies of a residential area of Dubai, UAE

### Tasneem Sandozi, Aksha Memon, Maira Khalid Mehmood, Nayyab Mohammed Tayyab Mustafa, Almas Parker, Kawther Hamdi

#### Dubai Medical College, Dubai, UAE

**Aim**

To determine the drug utilization of over the counter (OTC) drugs in private pharmacies of a residential locality of Dubai, UAE.

**Material & Methods**

A cross sectional prospective study was done over a period of two months in a residential locality of Dubai after obtaining prior approval from the authorities of the area pharmacies. The customers procuring OTC drugs were noted during this period and were briefly interviewed related to a short questionnaire prepared for this study. The questionnaire covered demographic and medical details of the customers, complete information of the purchased drug and also gave an insight on the customer’s awareness and knowledge of the OTC drug. Furthermore it was also taken into consideration whether the drugs were dispensed by the pharmacist; self-medicated by the patients or procured by presenting an earlier prescription.

**Results**

It was observed that non-steroidal anti-inflammatory drugs (NSAIDs) (62.85 %) and antihistaminics with decongestants (17.14 %) constituted the major groups of drugs sold OTC in the area of our study. Paracetamol (72.7 %), was the predominant NSAID dispensed OTC followed by ibuprofen (9.09 %), acetaminophen (9.09 %) and salicylates (9.09 %). Cetirizine (33.33 %), was the most commonly prescribed antihistaminic followed by loratidine (16.67 %), diphenhydramine (16.67 %) and amongst the nasal decongestants xylometazoline drops and pseuoephedrine were prescribe equally (16.67 %). Amoxicillin and terbinafine were the two antimicrobials dispensed OTC to the same extent (50 % each) of the antimicrobial drugs which were prescribed to an extent of 5.17 % of all he OTC drugs. The miscellaneous group of OTC drugs included various drugs like antihypertensives (telmisartan), antisecretory (pantaprazole), vitamin supplements and topical eye medications. The method of dispensing was self-medicated (83 %), pharmacist dispensed (14 %) and with an earlier prescription (3 %).

**Conclusion**

It is a general consideration that OTC drugs are absolutely safe without any risk of adverse effects. Non-steroidal anti-inflammatory drugs (NSAIDs) and antihistaminics, the two commonly used OTC drugs in this study have the potential of serious adverse outcomes and interactions that could be fatal. It should be the responsibility of the pharmacists to warn the recipients of the potential dangers while dispensing OTC medications.

## P7 General education matters for envisioning holistic integrative thinking in healthcare professionals

### Sabeena Salam

#### Dubai Pharmacy College, Dubai, UAE

**Background**

The General Education (GE) program is anchored in the recognition that students will live and work in a changing, often unpredictable world [1]. Around the world, general education, sometimes labeled liberal studies, has taken center stage as core curriculum requirement. In the UAE, GE reorganization and curricular reforms constituted the primary goal of the Commission of Academic Accreditation (CAA), Ministry of Higher Education and Scientific Research (MoHESR) [2]. In recent times, in this context, the GE programs have been able to move beyond course-integrated instruction into a formal planning role for general education programmatic offerings. There are good many reasons why general education plays an essential role in the baccalaureate degree. The poster shows the feasibility and benefits of such a program-offering in the context of health professionals’ education.

**Designs**

Given the variability of careers and employment, together with the various unforeseen directions that personal development may take, GE is dedicated to students' need for both the necessary capacities for life-long learning and a knowledge base that is transferable across academic disciplines and vocational (health care) contexts [3].

**Results**

Consequently, it provides students with the opportunity to enhance their ability to think critically; develop their quantitative reasoning, communication skills, technology and information literacy, ethical and moral reasoning; stimulate their capacities for creative, innovative thinking, and enrich their knowledge of the wider social, cultural, and natural worlds in which they will have to live and work.

**Conclusion**

Thus, the GE curriculum strives to provide a basis on which students can develop awareness of the intellectual, moral, aesthetic, and social contexts of their lives which is vital to 21st century healthcare professionals.

**Keywords:** general education, healthcare professionals

**References**

1. Mont Royal University, Retrieved from https://www.mtroyal.ca/AboutMountRoyal/TeachingLearning/GeneralEducation/TheImportanceofGeneralEducation/index.htm The Importance of General Education /index.htm, Accessed on April 27, 2015.

2. Standards for Licensure and Accreditation (2011), Commission for Academic Accreditation, Ministry of Higher Education and Scientific Research, United Arab Emirates. Retrieved from https://www.caa.ae/caa/images/Standards2011.pdf. p. 11–12.

3. Extracts from the Qualifications Framework Emirates Handbook and Implementation of QF Emirates in Higher Education September 2012, Retrieved from https://www.caa.ae/caa/.%5Cimages%5CQFEmirates_HB.pdf. Accessed on May 2, 2015

## P8 Course report: multidimensional imaging and capturing the quality of offered courses

### Eman Abu-Gharbieh^1^, Sabeena Salam^2^

#### ^1^Department of Pharmacology and Toxicology, Dubai Pharmacy College, Dubai, UAE; ^2^Dubai Pharmacy College, Dubai, UAE

##### **Correspondence:** Eman Abu-Gharbieh - Department of Pharmacology and Toxicology, Dubai Pharmacy College, Dubai, UAE

Of late, with the increasing need for mandatory accreditation, there have been concerted efforts in enhancing health educational programs by enriching the curricula content, teaching pedagogy and assessment methodology. In order to measure the effectiveness of the offered program, various key performance indicators (KPIs) should be assessed. Considering the complexity of healthcare education, a multi-source continuous feedback system has brought a paradigm shift in assessing performance and quality of a course offered. Courses are considered the corner stones in any academic program. Accordingly, Dubai Pharmacy college developed a comprehensive course report form in order to give a full imaging regarding the course efficacy including: course learning outcomes alignment with program objectives, course coverage, matrix mapping of assessment tools with learning outcomes, quantitative analysis of student performance, students feedback on the course, planning for improvement and finally the program review and assessment committee consensus that includes the assessment of the appropriateness of balance of assessment, relevance between the course contents and exams, appropriateness of prerequisites /co-requisites and text books. Contrary to a single instructor’s adjudication, this system evaluates an overall performance from comments received from learners, colleagues, supervisors as well as by a self-assessment, thereby minimizing prejudice in assessment. The system captures a ‘multidimensional image’ of the course taught and can even augment instructors’ professional developmental needs. However, the system of collating such multi-tier feedback appears to have its own flip-side. Yet, program managers consider multi-source feedback data as a valuable input for managerial decision making and policy development.

**Keywords:** KPI, learning outcome, pedagogy, course report.

## P9 Recent advances in medical education

### M. Jawad Hashim

#### Department of Family Medicine, UAE University, Al Ain, UAE

**Introduction**

Research in medical education is advancing at an unprecedented pace. For clinicians as well as full time academics, keeping up with recent advances has become more demanding. Yet remaining up to date is crucial for teaching to be grounded in current research and recommendations. For these reasons, a synopsis of recent advances in medical education is presented.

**Methods**

Published peer-reviewed literature on medical education was reviewed on MEDLINE PubMed and Google Scholar for the past five years, from 2010 to 2015, using keywords and MeSH headings for knowledge, learning, and medical education. Original research articles reporting on relevant issues to medical education as well as articles on research methodology were reviewed. Highly cited articles were identified to discern emerging trends in the field.

**Results**

New developments in core teaching methods and assessment especially in clinical competency evaluation for safety in patient care have been reported. Simulation-based medical education is increasingly cited for mastery learning in a controlled preclinical environment prior to supervised patient care. Controversies in simulation fidelity, use of problem based learning and inculcation of professionalism continue to mire the literature. Future directions in medical education such as evaluating physician performance and patient outcomes have been highlighted.

**Conclusions**

Recent advances in medical education necessitate changes to traditional approaches to undergraduate and postgraduate medical education. Medical teachers should align their teaching and assessment methods to be evidence-based and grounded in theory while adapting to regional needs.

